# 

**Published:** 1906-03-10

**Authors:** 


### NOTICE TO CORRESPONDENTS.

All MSS., Letters, Books for Review, and other matters intended for the Editor should be addressed The Editor, "The Hospital," The Hospital Buildings, 28 & 29 Southampton Street, Strand, W.C.

The Editor cannot undertake to return rejected MS., even when accompanied by stamped directed envelope.

**Notice to Advertisers and Subscribers.**

All Advertisements, Orders for Copies of The Hospital, and Business Communications should be addressed to **THE MANAGER (Not to the Editor),** The Hospital, 28 & 29 Southampton Street, Strand London, W.C.

The Cost of Subscription, post free, is as follows:—Per week, 3½d.; per quarter, 3s. 3d.; per half-year, 6s. 6d.; per annum, 13s. Foreign and Colonial:—Per annum, 19s. 6d., payable, in all cases, in advance.

**NOTICE.—Vol. XXXVIII. of "The Hospital" April to September 1905), in handsome cloth binding, will shortly be on sale at the office of this Journal, price 10s. 6d. Cases for binding the Numbers contained in this Volume 2s. Index to Vol. XXXVIII., 3½d., post free.**

**The Monthly Part for March is now ready. Price 1s., by post, 1s. 3d.**

### BOOKS RECEIVED.

H. K. Lewis.

"Massage and the Swedish Movements." 6th edition. By K. W. Ostrom.

Longmans and Co.

"The Health of our Children in the Colonies." By Dr. Lilian A. Robinson.

Macmillan and Co., Limited.

"The Archives of the Midlesex Hospital." Volume VI.

J. Murray.

"Chemistry of the Albumens." By S. B. Schryver.

A. and C. Black.

"Rome." By Eustace Reynolds-Ball.
"Who's Who, 1906."
"Who's Who Year-book, 1906."
"Black's Medical Dictionary."

Baillière, Tindall and Cox.

"Gynæcological Diagnosis." By A. E. Giles, M.D.
"Surgical Diseases of the Dog and Cat." By F. Hobday.
"Maladies Caused by the Air we Breathe." By Thomas Oliver.
"Treatment of Gonorrhœa." By C. Leedham-Green.

Kegan Paul, French, Trubner and Co.

"Nutrition and Dysentery." By U. N. Mukerji, M.D.

British Sports Publishing Co., Limited.

"Muscle Building."
"How to Wrestle."
"Dumb Bells."
"How to Punch the Ball."

W. B. Clive.

"Matriculation Directory." No. XLII. January, 1906.

Oliver and Boyd.

"Notes on Surgery for Nurses." By Joseph Bell, M.D.

A. C. Fifield.

"Curdled Milk, a Natural Key to Health and Long Life." By Dr. Montenius.

P. S. King and Son.

"Women as Barmaids."

The Scientific Press, Limited.

"Medical Homes for Private Patients."

***NOW READY.***

**BURDETT'S**

**Hospitals and Charities, 1906.**

The Year Book of Philanthropy and Hospital Annual.

**Price 6/- net; 6/5 post free.**

THE SCIENTIFIC PRESS, Ltd.,
28 & 29 Southampton Street, Strand, London, W.C.

### HOW TO BECOME A NURSE

**Full particulars of Training Schools, Premiums, Salaries, Hours of Duty, and all other details required by anyone desirous of entering the Nursing Profession will be found in**

**THE NURSING PROFESSION:**
**How and Where to Train.**

By Sir HENRY BURDETT, K.C.B.

Price 2/- net, 2/4 post free.

**It is essential that anyone desirous of taking up Nursing should have some idea of the duties of a Nurse.**

**NURSING HINTS TO PROBATIONERS ON PRACTICAL WORK.**

By ANNESLEY VOYSEY. Price 2/- net. 2/4 post free.

**Contains full particulars of a Nurse's duties and will prove of the greatest assistance to the Probationer.**

### Some Books indispensable to the Nurse Probationer

Dictionary of Terms used in Medicine, Midwifery, and Nursing.

**THE NURSES' PRONOUNCING DICTIONARY.**

By HONNOR MORTEN. New Edition, Revised by MARY I. BURDETT. 2/- post free.
This Dictionary has been used by Nurses for many years, and the fact that it has been recently revised and the phonetic pronunciations added by Miss M. I. Burdett should greatly enhance its value as a work of reference. Its size enables it to be carried comfortably in the apron pocket, which is a strong point in its favour.

How to Bandage.

**PRACTICAL GUIDE TO SURGICAL BANDAGING AND DRESSINGS.**

By WM. JOHNSON SMITH, F.R.C.S. 2/- post free.
A useful guide to Bandaging and Dressing, written especially for Nurses by a surgeon of great experience. The work is up to date, and the information thoroughly reliable.

Anatomy and Physiology.

**ELEMENTARY ANATOMY AND SURGERY FOR NURSES.**

By WILLIAM McADAM ECCLES, M.S.Lond., M.B., F.R.C.S., L.R.C.P. 2/6 post free.

**ELEMENTARY PHYSIOLOGY FOR NURSES.**

By C. F. MARSHALL, M.D., B.Sc., F.R.C.S. Crown 8vo, Illustrated, cloth. 2/- post free.
A valuable help to Physiology for Probationers.

Medical and Surgical Nursing.

**A HANDBOOK FOR NURSES.**

By J. K. WATSON, M.D., M.B., C.M. New and Revised Edition. Profusely Illustrated. 5/- net, 5/4 post free.

**SURGICAL INSTRUMENTS AND APPLIANCES USED IN OPERATIONS.**

A Classified and **Illustrated** List, with Explanatory Notes. By HAROLD BURROWS, F.R.C.S. Ready Shortly. Limp cloth. 1/6 net, 1/8 post free.
**This work will prove of the utmost service as a work of reference to all Nurses, as not only does it contain particulars of instruments, but is fully illustrated so that they can be readily recognised.**

Medical Electricity.

**MEDICAL ELECTRICITY AND THE LIGHT TREATMENT.**

By KATE NEALE, Sister in Charge Light Department, Guy's Hospital.
Cloth, profusely Illustrated, 2/6 net, 2/9 post free.
A practical book on a subject hitherto untouched from a nursing standpoint.

A Complete Text-Book of Medical, Surgical, and Monthly Nursing.

**NURSING: ITS THEORY AND PRACTICE.**

By PERCY G. LEWIS, M.D., M.R.C.S., L.S.A. New Edition Profusely Illustrated. 3/6 post free.

Surgical Work.

**SURGICAL WARD-WORK AND NURSING.**

By ALEXANDER MILES, M.D.Edin. C.M., F.R.C.S.E. Second Edition. With particulars and Illustrations of the Instruments used in various Operations.
3/6 net, 3/10 post free.

**THE EXAMINATION OF THE URINE.**

Compiled for the use of Nurses. By J. K. WATSON, M.D. M.B., C.M. 1/- net, 1/1 post free.

Write for Complete Catalogue, which will be sent post free on application.

**The Scientific Press, Ltd.,**

**Publishers of Nursing Text-Books, Charts, and Case-Books of all Descriptions.**

**28 & 29 SOUTHAMPTON STREET, STRAND, LONDON, W.C.**

